# Co-production for or against the university: student loneliness and the commodification of impact in Covid-19

**DOI:** 10.1108/QRJ-02-2021-0016

**Published:** 2021-06-22

**Authors:** Fred Cooper, Charlotte Jones

**Affiliations:** Wellcome Centre for Cultures and Environments of Health, University of Exeter, Exeter, UK

**Keywords:** co-production, engagement, loneliness, mental health, higher education, Covid-19, care, pandemic, diaries, neoliberalism

## Abstract

**Purpose:**

This paper explores the dissonance between co-production and expectations of impact in a research project on student loneliness over the 2019/2020 academic year. Specific characteristics of the project - the subject matter, interpolation of a global respiratory pandemic, informal systems of care that arose among students, and role of the university in providing the context and funding for the research - brought co-production into heightened tension with the instrumentalisation of project outputs.

**Study design/methodology/approach:**

Our project consisted of a series of workshops, research meetings, and mixed-methods online journalling between 2019-2020. This paper is primarily a critical reflection on that research, based on observations by and conversations between the authors, together with discourse analysis of research data.

**Findings:**

We argue that co-producing research with students on university contexts elevates existing tensions between co-production and institutional valuations of impact; that co-production with students who had experienced loneliness made necessary space for otherwise absent support and care; that our responsibility to advocate for our evidence and co-researchers came into friction with how the university felt our research could be useful; and that each of these converging considerations are interconnected symptoms of the ongoing marketisation of HE.

**Originality/value:**

This paper provides a novel analysis of co-production, impact, and higher education in the context of an original research project with specific challenges and constraints. It is a valuable contribution to methodological literatures on co-production, multidisciplinary research into student loneliness, and reflexive work on the difficult uses of evidence in university contexts.

## Introduction

Students living on the University of Manchester’s Fallowfield residential campus woke one Thursday morning to find that metal fences had been erected around their accommodation (The Guardian 2020). Without receiving any warning from the University, the students discovered on closer inspection that they were only able to leave the barriers through a single exit guarded by security. The fencing – at a cost of £11,000 – was said to be part of measures intended to minimise the spread of the coronavirus disease 2019 (Covid-19) during England’s November 2020 lockdown, by preventing access to the campus by non-residents (BBC News 2020a). Hours later, the University responded to student complaints by apologising, and announced they would remove the fences the following morning. However, later that evening, Fallowfield residents amassed to protest against the University’s response to the pandemic, including their inadequate mental health support and the unexpected installation of the fences. At the end of the rally, protesters pulled down the barriers that had been erected around their halls of residence, and trampled them to the ground.

This particularly visible episode crystallised longer and deeper tensions between the actions and policy of higher education (HE) institutions, including in response to the Covid-19 pandemic; students’ own self-advocacy; and contested public narratives on student loneliness and mental health, already persistently commodified and euphemised under the banner of ‘student experience’ (Pötschulat, et al., 2020). This paper explores these tensions through critical reflection on a piece of co-produced research on student loneliness which began in the autumn of 2019, culminated in a collective journalling project with 14 participants over nine weeks from May to July 2020, and had an ongoing legacy in terms of drawing out useable insights for the host university, which was also the funder. The project resulted in part from a successful submission to an internal funding stream, and brought together the interests of several researchers working on or around loneliness at an interdisciplinary research centre at the university. The aim of the work was relatively loose in the first instance, within the broad parameters of co-creating evidence on students’ experiences of loneliness. Research distribution and the communication of findings were still ongoing at the time of writing, but the dissolution of the research group (the majority of students were leaving the university) left us as custodians and advocates for the evidence they co-produced. Our advocacy was and is rooted in this evidence, but also stems from our continued contextual interpretation of the experiences they shared with us.

The potential for neoliberalism to constrain, ‘capture, and domesticate’ the transformative potential of co-production is well attested (Bell and Pahl 2018: 107). This paper considers how these dynamics are reconstituted when the participants and community co-producers of the research are members of a university, alongside the researchers, and the research itself is based wholly within, and funded by, the University. We argue that this shared research location brings distinct, or elevated, concerns about power, extraction, and co-production, and the potential for research to be co-opted and instrumentalised within the neoliberal academy. Our research took place in a context where co-production methodologies are valued primarily for the richness of the evidence they are supposed to create, but where quick, relatively superficial, ‘care-less’ approaches (Lynch 2010) tend to be privileged, and where even the most robust presentation of evidence does not (and in some cases cannot) translate into policy change. The beginning of the 2020/2021 academic year was also a moment in which Universities’ duty of care to isolating students was publicly at stake due to public conflict over campus safety during the Covid-19 pandemic, and the experiences and welfare of our student co-researchers were invested with heightened potential for commodity. As we explore, in the process of co-creating research, students who had significant pre-existing experiences of loneliness made room for informal systems of care in ways which had been absent or inaccessible outside of the project. These systems of care amplified an increasing dissonance between experiences and evidence that were valued by the authors and co-researchers, and the vexed and instrumental process of providing expert advice to a neoliberal institution attempting to navigate urgent and interlocking biopolitical and psychosocial crises.

The aims of this paper are multiple. Firstly, it situates the project, and our analysis, in a series of interconnected historical contexts: the ambivalent status of co-production within HE; endemic loneliness and mental health difficulties among students, in part due to environments and conditions which universities have always played a role in cultivating and maintaining (Pötschulat, et al., 2020); public health responses to Covid-19 which rely on isolation and distance; and sector-wide efforts to direct and manage student populations. Secondly, it details how solidarity and care emerged not just as theoretical objects of research, but as reciprocal practices which began at the margins of the project. Thirdly, it brings these practices of care into collision with difficult questions about who co-produced research is for and how it is used (Smyth 2020) when it creates space for implicit or explicit critiques of the host institution.

## Co-production in the university

Despite the recent resurgence of interest in co-production from universities and funders, higher education in the current climate is understood to be more or less inhospitable to co-production and engaged methods (Bell and Pahl 2018, Poleykett and Heney, forthcoming, Williams et al. 2020). For example, we have been warned against the potential for neoliberalism to co-opt co-production in a way that dilutes, exploits, or represses the work, or ultimately turns it against those involved in its creation (Bell and Pahl 2018: 109). Thus, ‘inclusion within existing structures’ (Bell and Pahl 2018: 110) may not grant the freedom or resources required for this research to be executed effectively, and may not appeal to the communities and collaborators with whom we work. As Bell and Pahl note (2018: 110), these communities ‘may prefer to increase their own material power outside of such representational structures’.

The marketisation of higher education has led knowledge production to be captured and ranked in terms of ‘quality’, ‘impact’ and ‘reach’ (Bell and Pahl 2018), placing limitations on co-produced work and devaluing the benefits it may offer beyond these criteria. On these terms, Morley (2016: 28) observes that academic research ‘values income generation, commercialisation, mobilisation and performance management over creativity, criticality, discovery or scholarly independence’. The impact agenda embodies this conflicting neoliberal imperative for both market rationality and social responsibility (D’Aoust 2014). In pursuit of maximising research productivity and efficiency (Elliott et al., 2017: 573), for instance, the slow and careful collaborations developed through engaged research may be overlooked (Durie et al. 2012). Mason (2021) underlines the ethical importance of working at a pace tailored to collaborators’ needs and commitments, sometimes quick and other times radically slow, a tempo which he argues poses a challenge to institutional logic and priorities. However, researchers – especially those early in their career – may find it impossible to fully adopt engaged research practices within the timeframes they are given, with the inflexibility of fixed-term contracts and doctoral funding (Poleykett and Heney, forthcoming).

These barriers notwithstanding, co-production is still purported to bear resistive and radical potential both to further the aims of social justice (Facer and Enright, 2016) and to disrupt traditional research relations of power, exploitation and domination (Bell and Pahl 2018). Thus, for some, it is imperative that co-produced methods operate ‘against academia’ as a means of transforming higher education itself (Bell and Pahl 2018: 112-113). To this end, Bell and Pahl (2018: 112) advocate for working in collaboration with communities who will ‘rock the boat’ and contribute towards challenging the norms we seek to dismantle. Whilst critical or dissenting collaborators may be well-received and conducive to ambitious and radical work in some circumstances, distinct challenges emerge when – as in the research explored in this paper – collaborators are invited to critique the research institution itself, either overtly, or by articulating their distress within this context. The neoliberal university is one which views the student as ‘a rational consumer with preferences that should be met with market mechanisms’ (Potschulat, et al., 2020). In this work, the financialisation of university policies and culture not only impacts the co-productive possibilities available to us, but also impacts the experiences of our (student) collaborators, and potentially transforms our interpretation (as university staff) of the collaborators’ insight. Ahmed (2015) notes how, when students challenge the university, complain, or show dissatisfaction, they are dismissed as acting like consumers. Despite a consumer identity also being actively encouraged by HE institutions (Pötschulat, et al., 2020), accusations of consumerism, Ahmed argues, can be used to ‘sweep away’ their critique.

Engaged and co-produced research are often practiced with the understanding that this type of work must involve outreach or collaboration beyond or outside of the academy due to researchers’ limited perspectives (Poleykett and Heney, forthcoming), and scholarship on the benefits and challenges of co-production largely assumes a separate location for the researchers and ‘the researched’ community. This elides numerous variations of insider, peer, and user-led research, whereby researchers share a (sometimes undisclosed) positionality with the participants or communities with whom they work (Poleykett and Heney, forthcoming), as well as overlooking the importance of critical, collaborative research on and about the institutions we are based within. Working in partnership with students not only challenges established research-participant binaries through engaged practice, but also traditional student-staff hierarchies (Dwyer 2018). Whilst this way of working does not eliminate the unequal power distribution in either of these contexts, a nascent literature on ‘Students as partners’ (SaP) - which focuses on more collaborative approaches to teaching and learning, rather than co-research - argues that these relationships of reciprocity can provide an ‘alternative approach […] in the increasingly economic-driven neoliberal landscape of higher education’ (Matthews et al. 2018).

## Contexts

The themes that this paper explores are each products of complicated and tangled histories, which we do not have space to do full justice to here. Instead, we set out several key contexts, which frame our primary discussion and analysis.

### Student loneliness

Student loneliness sits at the intersection of two endemic structural harms. In the first instance, high rates of reported loneliness in British universities are one facet of a wider problem in relational health, with deep cultural, political, and historical roots (Vincent 2020, Taylor 2020). Over the last few years, children and young people have been increasingly constructed as vulnerable populations, within the important context of broader anxieties around loneliness across the life-cycle. Young people navigate a series of pressures, tensions, and transitions, particularly around changing relationships, identities, bodies, and expectations, alongside intimacy, belonging, and distance. They also inhabit specific political, cultural, and economic contexts, which often make isolation more likely (Batsleer and Duggan 2020). Within this construction, there is a pressing need for work which goes against the grain of ‘neoliberalising discourses and pedagogies in which issues such as loneliness are individualised and pathologised, with young people made responsible for being resilient to adversity’ (Duggan 2020). Although outside of this specific context, mature students frequently encounter additional barriers to belonging which increase the potential for loneliness to occur (Sutton 2019). The relational challenges common to most students – for example, the cultural pressure to quickly form lasting friendships in the first weeks of term, and to perform a certain type of carefree sociality – often frame serious experiences of loneliness, even before broader health and social inequalities are taken into account.

In the second instance, student loneliness is a porous component of widespread experiences of mental ill-health at university, experiences which have motivated increasing attention and concern from academics, students, families, institutions, well-being services, and accommodation providers. Depending on where and how they are produced, discourses on student mental health can be relatively comfortable and well-received, revolving around decontextualized and simplified discussions of complex and structural ‘challenges’ such as geographical and emotional transitions or student accommodation (Christie et al. 2005). At other times, they constitute overtly political critiques or complaints, and can be awkward, uncomfortable, or unwelcome. This is particularly likely when they foreground systemic social and health inequalities, excessive tuition fees, inadequate mental health support, and entrenched cultures of coloniality, racism, transphobia and other forms of exploitation/prejudice which, when exposed, often go inadequately addressed (Doharty et al. 2021; Valentine et al. 2009). Loneliness is one of many ways in which these traumas and pressures are translated into individual experience and feeling, but it also has a complex inter-causal relationship with depression, anxiety, frustration, unhappiness, and stress. Although itself open to critique, loneliness is presently the most recognisable language we have to articulate complicated and difficult feelings of isolation, estrangement, and abandonment (Ozawa de Silva and Parsons 2020: 614-615).

However these contexts and complexities are parsed, quantitative surveys over the last few years demonstrate that a troubling proportion of students felt acute or chronic loneliness in the course of their degree before the Covid-19 pandemic (WonkHE/Trendence 2019), gesturing to a longer inability within HE to foster environments where diverse student populations have access to good relational health. Austerity cuts and the larger neoliberal agenda have also eroded many of the mechanisms and services used to alleviate loneliness, most severely impacting those who were already struggling, such as poorer communities (Stenning and Hall 2018). Despite student loneliness and mental health having long been a key agenda for universities (Crook 2020), and rising demand for university counselling and support sectors, these have been significantly reduced and outsourced across the sector, with waiting lists of over four months in many institutions (Independent 2018; Jones and Cooper 2020). Our research with students, and their experiences of loneliness, disconnection and exclusion, began before Covid-19. Some students’ palpable relief at returning home – and dread at re-entering university life after restrictive public health measures end – illustrates longer temporalities of past and future loneliness which are frequently absent from crisis-oriented and presentist discussions of pandemic isolation (Cooper 2020).

### Covid-19 and Higher Education

The felt effects of public health responses to Covid-19 take place against this historical backdrop. Such responses are also novel for the majority of the population (albeit to varying degrees) in the isolation and distance that they impose. To minimise transmission, government guidance has functioned by limiting contact, keeping many people physically separate from those who care about them, and greatly reducing (or sometimes eliminating) significant experiences such as reciprocal touch. For many, the pandemic has put almost every aspect of life - and mental and relational health - under strain, exerting serious financial pressures, causing particular groups to feel unsupported or abandoned, transforming experiences of work, introducing infection anxieties to shared spaces, destabilising future-oriented planning and preparation, highlighting rifts in values and moral priorities, and creating new and dislocating iterations of grief and shame (Cornwall Live 2020, Covid-19 Bereaved Families for Justice UK 2020, Dolezal and Rose 2020, Hale 2020, Harrop et al. 2020, Independent 2020, Smith et al. 2020). Loneliness, neglect, and alienation have been acute and widespread in response to the pandemic and its political and social repercussions in the UK, even though much of this adversity already existed in some form for many people due to endemic social and structural inequalities (Rose et al. 2020).

In this fraught and brittle landscape, student loneliness and mental health have played key roles in public conversations about the responsibilities of higher education institutions during the pandemic. In 2020’s autumn term, institutions clung tightly to several aspects of university life which necessitate contact, particularly in-person (or ‘face-to-face’) teaching and accommodating students in halls of residence, despite pressure from the University and College Union (UCU), the leading British trade union for academics and related professional services staff (UCU 2020a) and the National Union of Students (NUS) (UCU 2020b). Whilst university executives have played a very limited role in public discourse, within more private internal communications online-only teaching has been condemned as a risk to student wellbeing and mental health, likely to lead to isolation and anxiety (University of Bristol, quoted in Bristol UCU 2020, University of Exeter 2020, University of Sussex 2020a), with in-person teaching presumed to minimise staff loneliness (University of Exeter 2020).

Protecting student mental health and mitigating loneliness have also been the primary justifications for Universities UK (UUK) (UUK 2020) and the UK government (University of Sussex 2020b) to retain on-campus working, despite the rise in Covid-19 cases brought by the return or arrival of students to their universities (see also Sussex UCU 2020, Jones and Cooper 2020). Before some universities had begun their Welcome Week in September 2020, others had started to self-isolate students. This was later the case for thousands of students at different universities across the country. One student noted that the measures would have ‘a big impact on the mental health of a lot of the students’. She added, ‘I just feel completely neglected; there’s been little in the way of pastoral care’ (BBC News, 2020b).

Each of these contexts are interconnected. The tensions between co-production, care, and the commodification of impact are products of the conditional success of a specific neoliberal imaginary of how universities should operate; the success of this imaginary is, in turn, implicated in students’ experiences of loneliness. The following section of this paper explores how co-production during the pandemic made space for both critique and care, detailing our methodological approach to the project, and analysing data which sheds light on how the project was experienced by our co-researchers. We then reflect on how we navigated uncomfortable assumptions that our work could be ‘useful’ to the university, in ways that had the potential to compromise our advocacy on behalf of our co-researchers and the evidence they produced.

## Care and Co-production

Our project began with a series of six in-person workshops in late 2019, each with between 1 and 7 participants (29 in total), followed by two online workshops during the first national Covid-19 lockdown in April 2020, with some of the same participants. In these eight workshops, we observed acts and extended moments of care, witnessing, disclosure, and affirmation in interactions between the students, some of whom would later become co-researchers. Students who made themselves potentially vulnerable to the group by speaking about difficult or stigmatised emotions and behaviours often had their experiences acknowledged with expressions of solidarity. Some students informed us that they benefited from the workshops in ways they had not necessarily anticipated; through listening to their peers and putting feelings of loneliness into narrative, and by sharing their own experiences in a sympathetic context. In making it clear that they valued the workshops and meetings as their only social space outside of the classroom, others implied that relational motivations were among their reasons for joining the project, looking to be part of an emerging community of purpose, or simply to enjoy socialising and making new friends (Duggan 2020).

The loosely-structured flow of conversation meant that students frequently emphasised the commonalities between their experiences and those of others, creating a sense of shared challenges. In discussing feelings such as loneliness, social anxiety, unhappiness, and disappointment, there was a consistent acknowledgement that they were emotionally dissonant, unmentionable, and neglected; both in ordinary interactions with other students and the institutional cultures and contexts which framed them. There was room for care in these workshops that was conspicuously absent in their other experiences of university, as well as an explicit desire to change the institution’s approach to loneliness, and to improve these circumstances for future students.

In these sessions, we explored ways of documenting how the pandemic was experienced by students with pre-existing experiences of loneliness, opening up opportunities for creativity and introspection, and allowing for dialogue to continue and flourish within the group. Whilst this felt beneficial to some, our student group also reduced in size at this stage, with some co-researchers sharing that they were otherwise preoccupied by the challenging circumstances, and unable to continue. Taking the project forward, one student suggested journalling, which was well-received; including by us, mindful of what kinds of work would translate into shareable and reportable outcomes within our specific professional and institutional context, as precarious researchers delivering on internal funding (Williams et al. 2020). In valuing co-production primarily as an approach which leads to ‘better’ and more impactful research, institutions exert pressure on university researchers to work in ways which have the potential to undermine the principles on which co-production operates, muddying the borders with more didactic or extractive methodologies and power relationships (Bell and Pahl 2018). Precarious researchers in particular rarely have time to engage in what can be difficult and devalued work, at least in planning and execution (Poleykett and Heney, forthcoming). This specific and partial valuation actively works towards slippage between co-production as a robust methodology and co-production as an aesthetic cover for research which is merely participatory or consultative (Williams et al. 2020).

We interpreted the idea of a journal relatively liberally (Jones, forthcoming), thinking particularly about the benefits of creative work, different styles of expression to make space for the multiplicities and diversities of loneliness (Duggan 2020), and the need to sustain the students’ interest when turning in weekly reflections. We created a private digital space in Microsoft Teams, where our 14 co-researchers could contribute a weekly response to different stimuli; a few written prompts or questions, and examples of a possible medium to try working in, such as comic strips, poetry, or art. Some students submitted every week whilst others dipped in and out, and our prompts were only loosely followed, with formal experimentation often carried on independently. Whilst no students withdrew entirely during the process, the journalling was intentionally flexible, and allowed for students to pause or temporarily disengage, depending on their circumstances, without causing any conflict. Alongside more conventional journals such as prose reflections or diaries, our co-researchers contributed photography, artwork, music, poems, films, letters, and cartoons. For each submission, the students received a payment in gratitude for their participation. These contributions were then visible to their peers (and to us), who could react with emojis, comment, and start conversations underneath one another’s posts. We made it clear that this kind of interaction was a potential use of the space (and we commented ourselves), but we wanted it to emerge as a decision made by the students, whether individually or collectively. Some students chose to respond to other people’s journal entries on weeks when they did not feel able to contribute an entry of their own. Alternative submission options were also provided, one for anonymous contributions and another that would only be viewed by the academic researchers, although these alternatives were used infrequently.

Within and around the rich seam of artistic and thoughtful texts on loneliness, public health, and Covid-19 that our co-researchers created, the supportive practices that characterised the early in-person workshops became a digital marginalia of care (Muddiman et al. 2018). In demonstrating the phenomenon we are referring to, we have chosen not to focus on the supportive comments themselves, although these were plentiful. Instead, we focus on comments which articulated how specific contributions affected readers/viewers, references to the process as a whole, and reflections from the final journal in week 9; moments where we gained a particular insight into how working on the project made our co-researchers feel. One exchange in the second week of the project crystallised a series of recurring themes:

Jessica: I think just sharing my narrative will help me to feel less isolated and help me to process what has happened in the last few months. (Reactions: heart emoji x6)

Sue: Jessica, you are so brave. As I read through your journal I felt as though I was with you in person.

Georgia: Thank you for sharing this, it was really brave of you! The way you described the whole experience really touched me (heart emoji). (Reactions: heart emoji x2)

Jessica: I can’t thank you guys enough for all your kind comments - very emotional for me! I really feel ‘seen’ and understood by you guys. I’m so glad I shared, I feel much less alone already (smiley face). (Reactions: heart emoji x2) (Jessica’s journal, week 2).

Jessica had uploaded her journal with two interlocking hopes; that communicating her experiences would lessen her feelings of loneliness, and that this would allow necessary introspective work to occur. In the conversation started by Jessica, we see Sue and Georgia expressing their solidarity in emotive terms, particularly given the dual contexts of their pre-pandemic experiences of loneliness, and the public health restrictions which necessarily imposed distance between them. They cast their emotional proximity as explicitly physical and present, at a historical moment where closeness and touch were a practical impossibility. This slippage and play between presence and absence, distance and immediacy, was also shown in comments by Olivia; both in her own writing and in a response to Wen’s journal, later in the project: ‘I felt like I was living in these moments with you’, ‘It means a lot that we have shared this space together through this time (heart emoji)’ (Wen’s Journal, week 3; Olivia’s journal, week 8).

When Jessica responded that she felt ‘seen’ and ‘understood’ by Sue and Georgia, she demonstrated a complexity of need which is frequently absent from simplistic constructions of loneliness as a straightforward lack of wished-for interaction. Her experiences of loneliness encapsulated an unmet need to communicate and connect, to share feelings and experiences and have them witnessed, internalised, and reflected back with knowledge and appreciation (Jessica’s journal, week 2). At every stage of the project, our co-researchers evidenced and articulated loneliness as complicated and contingent, requiring long-term, structural solutions which make room for genuinely meaningful connections and exchanges.

Sue followed Jessica’s cue on the importance of understanding in a later conversation:

Sue: Hang in there sweetheart. Jessica, because so much has been sent to try us over the past few months, it is only natural to feel anxious and out of sorts at the moment. I completely understand you, and just wanted to say, try to focus on what makes you feel good and I hope that this week gets better for you.

Jessica: I keep reading your comment and it makes me feel so much better, thank you Sue (Jessica’s journal, week 7).

Reflecting on the project in her final journal, Sue explored what giving care to her co-researchers had meant to her: ‘Even if I have been having a bad day, leaving something as simple as a smiley face or thumbs up on someone’s journal, helped me to feel that I was acknowledging that person and what they had to say’ (Sue’s journal, week 9). Loneliness often entails exclusion not just from being cared for, listened to, and understood, but from caring for, listening, and understanding others in turn (Seppala et al. 2013). Jessica concluded the exchange in week 2 with an emerging motif in how our co-researchers narrated their involvement with the project, writing explicitly that sharing with the group and receiving their responses made her ‘feel much less alone already’ (Jessica’s journal, week 2).

Articulated by different co-researchers at varying stages of the project, the recurring assertion that the process of co-producing this research was helping to alleviate the loneliness they experienced - the basis of their expertise and involvement - has important implications. As academics concerned with student loneliness as a broader ‘problem’, our co-researchers made this work immanent and personal; theirs were the experiences and perspectives we wanted to see valued and reckoned with. If our project was (temporarily) ameliorating loneliness and making room for care, this was a small-scale example of the overarching aim that we were working towards. This mattered to our research, and to us. When we communicate findings from our project, within our institution or outside it, co-production is an important component of our recommendations (Jones and Cooper 2020). We share the instrumentalised valuation of co-production as facilitating more effective engagement with the publics involved in its design (Hinchliffe et al. 2018). We also value it as able, in contrast with other methodologies, to build and sustain the more ethical, caring and supportive relationships and spaces which were present in our work. In co-producing evidence on student loneliness, our co-researchers were simultaneously creating evidence on co-production as an ameliorative methodology, where collaboration and dialogue constituted a form of care, whilst also addressing the students’ desire for institutional improvement. As the final section of this paper interrogates, that evidence had the potential to act as critique or complaint not just of pre-Covid-19 neoliberal universities, but of the loaded and visible actions of our host institution during a global pandemic, albeit in step with the vast majority of the sector. The awkward positioning of our work - at the university, on the university, and funded by the university - came with heightened and uncomfortable expectations of utility, which often felt out of alignment with the interests and experiences of our student co-researchers.

## Institutional Constraints

The journaling phase of the research drew to a close in late July 2020, a few weeks after England’s first lockdown measures were relaxed, and two months prior to students returning to their studies for the start of the new academic year. Around this time, our student co-researcher group also disbanded, with some students leaving the University or the area after completing their studies, and others wanting to focus on their final years of education. As pressures grew for eligible classes to be shifted entirely online and students to be discouraged from moving locations to their university residence after the summer break, the detrimental emotional and mental health consequences of ‘business as usual’ became progressively more evident. For weeks, international and local news headlines and social media platforms were saturated with outcries from distressed and dissatisfied students and their concerned parents. Protests followed; at the time of writing, student activists had organised rent strikes on at least 55 campuses across the UK, the largest of which – at Bristol University – included over 1,400 students (Tribune 2021). With this candid shaming and exposure of higher education (Smyth 2020), there emerged a threat that institutional reputations could be damaged.

As researchers of student loneliness, the perceived expedience and exigency of our work transformed rapidly. During the first UK lockdown in spring, we embraced this opportunity and provided an internal briefing to summarise our research findings and offer recommendations for the following academic year, including advice on the benefits of co-production in designing effective preventative measures for relatively predictable challenges around loneliness and mental health in the autumn term. Our institution later summoned our expertise, and invited us to provide input during the 2020/2021 autumn term, both privately in the decisions and consultations taking place amongst executives, as well as more publicly, to engage with the student body. Despite our investment in student welfare, we were unsure how to reconcile becoming ‘useful’ to the University at this time, only months after we had instigated this engagement. In arguing for urgent - and sustained – ameliorative measures, which require considerable allocation of resources alongside extensive structural and cultural change, we were keen to avoid working with the University in ways which were superficial, cosmetic, or face-saving. Our engaged loneliness work – designed and developed ‘with’ and ‘on’ students – was not intended (from our perspective) to provide a service, uncritically, *for* the University, and certainly not to defend or promote the University at a moment where their management of or care towards students was in question. However, this did not feel entirely within our control; regardless of our motivations and our dis/inclination to cooperate, the institutional affiliation and funding of this work could still be profitable for our employer. The tensions we outline here are not intended to form a commentary or critique on the actions, management, or culture of one university, but to draw out challenges held in common by critical researchers and practitioners of co-production techniques across the sector, and the (sometimes competing) pressures of an impact agenda.

The ethical and methodological underpinnings of our work – not only its focus – could also grant specific benefits to the University. Our interdisciplinary research serves as an example of students collaborating with academics over a period of months, developing trusting and sensitive relationships, and critically exploring social and relational practices within the institution. Poleykett and Heney (forthcoming) describe the bind of complicity in engaged research whereby, despite researchers seeing themselves as ‘working against’ the university, we are nevertheless always providing *for* the university, and therefore acting in the university’s interests. Although as researchers, we may see our work as located ‘beyond’ (and to some extent ‘against’) some of the constraints and directorate of the University, our student co-researchers are nevertheless fundamental components of the institution, as are we as employees. No matter how critical our work may be, we are still acting ‘within’ the University and its political culture; we are (part of) it.

The dilemma this presents is not exceptional. Doharty et al. (2020) observe the paradox of working as scholars of colour under a ‘decolonial’ agenda, whereby calls to decolonise universities have only entrenched whiteness further. They argue decolonisation provides a way for HE to demonstrate commitment to anti-racism, but in ‘exploitatively draining the useful parts of [BME academics’] scholarship to meet institutional metrics and marketise fashionable buzz-words’, they advance rather than dismantle racist practice (Doharty et al. 2020: 241). This can lead to BME scholars becoming complicit in ‘supporting the limited progress institutions herald as significant achievements’ (241). Thus, academics risk sustaining a broken system by producing research and knowledge which can be used in its defence. This is perhaps especially frustrating in cases where research provides incisive evidence of the damage that it can then be used to mask. In this project, for example, the students’ attraction and attachment to the temporary research space and the community we created was gratifying, but this itself was an indication that the kind of support and conversation we fostered was severely lacking elsewhere. Poleykett and Heney (forthcoming) note that in some instances engaged researchers produce exemplary work that the university can claim as ‘case studies and flagship projects without meaningfully addressing the way in which their own structures actively act in opposition to such work’. It was, of course, vital that this project was understood as a spotlight upon existing issues and a call for change, rather than a solution to them in itself. This was only a small project led by researchers on precarious contracts. Fundamentally, our work with students could not – and never intended to – provide a long-term solution for student loneliness; however, it did begin to reveal the absent mechanisms of care that were desperately needed.

The University’s increased interest in making use of our research also exposed some of the tensions and responsibilities behind the ownership and interpretation of academic work. The heterogeneous moments of struggle, distress, and loneliness that were expressed by students in their journals could be characterised in various different ways. After our engagement with the student collaborators had ended, we felt accountable to the collaborators to approach the data with care, whilst knowing that, as researchers, our power to interpret, analyse, and categorise are never neutral, and our ability to faithfully understand and make sense of the students’ contributions – as in all research – inevitably poses problems (Acker et al. 1983). Indeed, every stage of the research was relational; our presence, and the students’ familiarity with us, our motivations, and convictions also shaped their participation. Through these sustained, reciprocal, and caring relationships we formed with our co-researchers, we hoped to approach the data with a fuller appreciation of a context which may escape an outsider’s interpretation (Ceglowski 2000).

## Conclusion

The interpretations and conclusions we draw from research have critical ideological significance. We have observed how self-interest, economic imperatives, and opportunism drove some mental health discourses on the impact of the pandemic and subsequent lockdowns, particularly in circumstances where concerns about potential mental distress could be used to advance or defend other agendas. The assumed relationship between student loneliness, mental ill-health and government-enforced lockdowns was a persistent justification for in-person teaching, even though there was no reason to be confident that teaching in classrooms during the pandemic would resolve this loneliness, or that it would counterbalance other potential mental and physical harms that in-person teaching introduces to both students and staff. Sussex UCU (2020) rightly observed that in-person teaching has not protected students from increasing levels of stress, isolation and loneliness over the last decade. The promise of in-person teaching did, however, secure the movement of students to campuses at the start of the year, protecting universities’ home and overseas student fee and rent revenue (Holmwood 2020), which - in lieu of meaningful and unqualified crisis support from the government - was financially critical.

Our research with students who had experienced loneliness made necessary space for otherwise absent support and care. However, as we have illustrated, co-producing research with students about university experiences elevates existing tensions between co-production and institutional valuations of impact, and our responsibility to advocate for our evidence and for the co-researchers came into friction with how the university felt our research could be useful. Whilst all research is expected and intended to be impactful, co-produced research usually involves a specific accountability to the communities involved. When these communities are attached to the research institution, competing interests may emerge. Each of these converging considerations are also interconnected symptoms of the ongoing marketisation of HE. The same deep-seated structures position students as consumers, commodify their education, strip back and work against important determinants of relational health, and frame how co-produced evidence on the felt effects of these processes is instrumentalised in institutional decision-making. Much like community practices of care through mutual aid which grew in response to Covid-19 lockdowns, support was enacted spontaneously by students when their welfare was threatened. Mutual aid – and, indeed, the care the students provided for each other – is compatible with neoliberal logics when co-opted to justify or mask the harms to which communities were subjected, or to restore a sense or ‘normality’ (Firth, 2020: 57). However, these co-produced sites of care and solidarity can also develop opportunities for more autonomous critique of the contexts we work within, which are resistant - and perhaps directly confrontational to - the frameworks of marketisation that contribute to and shape the problem of loneliness.

Our experiences of co-production in a university context were and are ambivalent; the university funded our work on the understanding that co-production could generate ‘better evidence’ on student experiences of loneliness. In so doing, it also sets distinct institutional boundaries on the transformative potential of the resulting knowledge. Commodification and constraint are not alien to an imagined ‘perfect’ iteration of university-based co-production, they are its preconditions; amenable to mitigation and resistance but not, in current circumstances, to extrication. Despite this, neoliberal universities can sometimes allow scope for co-produced knowledge which challenges or unsettles environments and conditions which they have always played a role in cultivating and maintaining (Pötschulat et al. 2020). When institutions instrumentalise co-production, the extent to which it can be used as a methodology to shift the conditions of its own constraint are severely limited. Whilst these are not the only constraints upon engaged research practices, we suggest that if universities can open themselves up to the radical vulnerabilities that co-production can imply, and allow themselves to be led to dissonant or uncomfortable places, then co-produced knowledge which focuses on structural harms in higher education, and which advocates for systemic change, could begin to be meaningfully addressed.

In this paper, we have articulated our concerns about an instrumental and seemingly transitory interest in student loneliness, which we suggest has been taken up by some higher education executives during the Covid-19 pandemic. Student loneliness was, however, a pressing public health issue before Covid-19. When the global virological crisis recedes, the social and relational legacies of the pandemic – and how students experience, navigate, and interpret them – will frame new contexts for loneliness in HE. These legacies are likely to complicate, heighten and alter long-standing health inequalities, and produce fresh tensions between marketisation and care. The extent to which British universities, as presently constituted, are willing and able to work with students - through academic co-production or otherwise - to conceive and create radically different futures is very much in doubt.

## References

[R1] Acker J, Barry K, Esseveld J (1983). Objectivity and truth: Problems in doing feminist research. Women’s Studies International Forum.

[R2] Ahmed S (2015). Against Students. The New Inquiry.

[R3] Batsleer J, Duggan J (2020). Young and Lonely: The Social Conditions of Loneliness.

[R4] BBC News (2020a). New lockdown: Manchester University fencing costing £11k removed.

[R5] BBC News (2020b). Covid: Manchester Metropolitan students ‘feel completely neglected’.

[R6] Bell DM, Pahl K (2018). Co-production: towards a utopian approach. International Journal of Social Research Methodology.

[R7] Bristol UCU (2020). ‘Update: University Response to our Bristol UCU Motion‘.

[R8] Ceglowski D (2000). Research as Relationship. Qualitative Enquiry.

[R9] Christie H, Munro M, Wager F (2005). Day students’ in higher education: widening access students and successful transitions to university life. International Studies in Sociology of Education.

[R10] Cooper F (2020). COVID-19 and the Loneliness Crisis. Solitudes Past and Present blog.

[R11] Cornwall Live (2020). Cornwall restaurant staff reveal ‘horrific’ abuse from Eat Out to Help Out diners.

[R12] (2020). Covid-19 Bereaved Families for Justice UK.

[R13] Crook S (2020). Historicising the “Crisis” in Undergraduate Mental Health: British Universities and Student Mental Illness, 1944-1968. Journal of the History of Medicine and Allied Sciences.

[R14] D’Aoust A-M (2014). Ties That Bind? Engaging Emotions, Governmentality and Neoliberalism: Introduction to the Special Issue. Global Society.

[R15] Doharty N, Madriaga M, Joseph-Salisbury R (2021). The university went to ‘decolonise’ and all they brought back was lousy diversity double-speak! Critical race counter-stories from faculty of colour in ‘decolonial’ times. Educational Philosophy and Theory.

[R16] Dolezal L, Rose A (2020). Naming and Shaming: Covid-19 and the Medical Professional. Medical Humanities blog.

[R17] Duggan J (2020). The co-productive imagination: a creative, speculative and eventful approach to co-producing research. International Journal of Social Research Methodology.

[R18] Durie R, Lundy CA, Wyatt K (2012). Researching with Communities: Towards Leading Edge Theory and Practice for Community Engagement.

[R19] Dwyer A (2018). Toward the formation of genuine partnership spaces. International Journal for Students as Partners.

[R20] Elliott S, Ngo McKelvy J, Bowen S (2017). Marking time in ethnography: uncovering temporal dispositions. Ethnography.

[R21] Facer K, Enright B (2016). Creating living knowledge: The connected communities programme, community–university partnerships and the participatory turn in the production of knowledge.

[R22] Firth R, Preston J, Firth R (2020). Coronavirus, Class and Mutual Aid in the United Kingdom.

[R23] Hale J (2020). Shielders Have Been Abandoned During Lockdown - It Can’t Happen Again.

[R24] Harrop E, Farnell D, Longo M, Goss S, Sutton E, Seddon K, Nelson A, Byrne A, Selman LE (2020). Supporting people bereaved during COVID-19: Study Report 1.

[R25] Hinchliffe S, Jackson MA, Wyatt K, Barlow AE, Barreto M, Clare L, Depledge MH, Durie R, Fleming LE, Groom H, Morrissey K (2018). Healthy publics: enabling cultures and environments for health. Palgrave Communications.

[R26] Holmwood J (2020). UK Universities and Covid-19: Time for Cooperation, not Competition.

[R27] Independent (2018). Students wait up to four months for mental health support at UK universities.

[R28] Independent (2020). Coronavirus: ‘Onslaught’ of tenants will be unable to afford rent as research shows 1.7m believe they could lose jobs.

[R29] Jones C, Walker M Interdisciplinary and Global Perspectives on Intersex.

[R30] Jones C, Cooper F (2020). Covid-19 has amplified student loneliness and distress.

[R31] Lynch K (2010). Carelessness: A Hidden Doxa of Higher Education. Arts and Humanities in Higher Education.

[R32] Mason W, with Unity Gym Project (2021). Temporality in Qualitative Inquiry: Theories, Methods & Practices.

[R33] Matthews K, Dwyer A, Russell S, Enright E (2019). It is a complicated thing: Leaders’ conceptions of students as partners in the neoliberal university. Studies in Higher Education.

[R34] Morley L (2016). Troubling intra-actions: gender, neo-liberalism and research in the global academy. Journal of Education Policy.

[R35] Muddiman E, Lyttleton-Smith J, Moles K (2019). Pushing back the margins: power, identity and marginalia in survey research with young people. International Journal of Social Research Methodology.

[R36] Ozawa-de Silva C, Parsons M (2020). Toward an anthropology of loneliness. Transcultural Psychiatry.

[R37] Pötschulat M, Moran M, Jones P (2020). ‘The student experience’ and the remaking of contemporary studenthood: A critical intervention. The Sociological Review.

[R38] Poleykett B, Heney V ‘Complicity and accountability in engaged research’. Sociology of Health and Illness.

[R39] Rose N, Manning N, Bentall R, Bhui K, Burgess R, Carr S, Cornish F, Devakumar D, Dowd JB, Ecks S, Faulkner A (2020). The social underpinnings of mental distress in the time of COVID-19 - time for urgent action. Wellcome Open Research.

[R40] Seppala E, Rossomando T, Doty JR (2013). Social Connection and Compassion: Important Predictors of Health and Well-Being. Social Research.

[R41] Smith L, Duffy B, Moxham-Hall V, Strang L, Wessely S, Rubin GJ (2020). Anger and confrontation during the COVID-19 pandemic: a national cross-sectional survey in the UK. Journal of the Royal Society of Medicine.

[R42] Smyth J (2020). Critical social science as a research methodology in universities in times of crisis. Qualitative Research Journal.

[R43] Stenning A, Hall SM (2018). On the Frontline: Loneliness and the politics of austerity, Discover Society.

[R44] Sussex UCU (2020). In-person teaching during the pandemic: is it really about student mental health?.

[R45] Sutton CE, Boeren E, James N (2019). Being an Adult Learner in Austere Times.

[R46] Taylor B (2020). Solitary citizens: the problem of loneliness.

[R47] The Guardian (2020). Manchester students pull down lockdown fences around halls of residence.

[R48] Tribune (2021). Britain’s Historic Wave of Student Rent Strikes.

[R49] UCU (2020a). ‘Universities must not become the care homes of a Covid second wave’.

[R50] UCU (2020b). ‘UCU and the National Union Students issue joint statement calling for action over university Covid crisi’s.

[R51] Universities UK (2020). Universities UK response to UK government lockdown guidance for universities.

[R52] University of Exeter (2020). Message from the Vice-Chancellor for staff - 22 October 2020.

[R53] University of Sussex (2020a). View from the VC.

[R54] University of Sussex (2020b). Message from the UK Government to all students.

[R55] Valentine G, Wood N, Plummer P (2009). The experience of lesbian, gay, bisexual and trans staff and students in higher education.

[R56] Vincent D (2020). A History of Solitude.

[R57] Williams O, Sarre S, Papoulias SC, Knowles S, Robert G, Beresford P, Rose D, Carr S, Kaur M, Palmer VJ (2020). Lost in the shadows: reflections on the dark side of co-production. Health Res Policy Sys.

[R58] WonkHE (2019). Only the lonely - loneliness, student activities and mental wellbeing at university.

